# Is it possible to stabilize a thermophilic protein further using sequences and structures of mesophilic proteins: a theoretical case study concerning DgAS

**DOI:** 10.1186/1742-4682-10-26

**Published:** 2013-04-10

**Authors:** Ming Liu, Hongqiu He, Jiguo Su

**Affiliations:** 1 Institute of Materia Medica, Chinese Academy of Medical Sciences & Peking Union Medical College, Beijing, 100050, China; 2 Chongqing Center for Biomedicines and Medical Equipment, Chongqing Academy of Science and Technology, Chongqing, 401123, China; 3 College of Science, Yanshan University, Qinhuangdao, 066004, China

**Keywords:** Thermostability, Amylosucrase, Molecular modeling, Protein design

## Abstract

Incorporating structural elements of thermostable homologs can greatly improve the thermostability of a mesophilic protein. Despite the effectiveness of this method, applying it is often hampered. First, it requires alignment of the target mesophilic protein sequence with those of thermophilic homologs, but not every mesophilic protein has a thermophilic homolog. Second, not all favorable features of a thermophilic protein can be incorporated into the structure of a mesophilic protein. Furthermore, even the most stable native protein is not sufficiently stable for industrial applications. Therefore, creating an industrially applicable protein on the basis of the thermophilic protein could prove advantageous. Amylosucrase (AS) can catalyze the synthesis of an amylose-like polysaccharide composed of only α-1,4-linkages using sucrose as the lone energy source. However, industrial development of AS has been hampered owing to its low thermostability. To facilitate potential industrial applications, the aim of the current study was to improve the thermostability of *Deinococcus geothermalis* amylosucrase (DgAS) further; this is the most stable AS discovered to date. By integrating ideas from mesophilic AS with well-established protein design protocols, three useful design protocols are proposed, and several promising substitutions were identified using these protocols. The successful application of this hybrid design method indicates that it is possible to stabilize a thermostable protein further by incorporating structural elements of less-stable homologs.

## Introduction

Life flourishes almost everywhere on earth, from hydrothermal vents in the deep-sea to the tops of the Himalayas, from rain forests to the hot sands of the Sahara desert, and even from the boiling waters of hot springs to the cold ice field of Antarctica. Organisms that inhabit such harsh environments must evolve to adapt those living conditions. In relation to temperature adaptations, environmental stress generally cannot be avoided through compensatory mechanisms, as is the case for other types of adaptations [1]. Therefore, cellular and cytoplasmic components, specifically proteins, must achieve thermostability [2]. For this reason, much effort has been directed towards understanding how proteins from thermophilic organisms retain their structure and function at elevated temperatures [3-7]. Such understanding is essential for a theoretical description of the physicochemical principles underlying protein folding and stability, but it is also critical for designing proteins that can work at high temperatures or are more resistant to unfolding at certain working temperatures. High thermostability is required for several industrial applications including detergent manufacturing, food and starch processing, production of high fructose corn syrup and PCR [8-10]. Furthermore, thermophilic proteins are more resistant to proteolysis and chemical denaturation than their mesophilic homologs [11]. In general, thermophilic proteins possess multiple features that are important for high thermostability including more hydrogen bonds and salt bridges, and higher contact order, than their mesophilic counterparts. In view of this, much research has focused on elevating the thermostability of mesophilic proteins through investigating the features of their thermophilic homologs. By substituting key residues or even motifs on the basis of the sequences of homologs, the thermostability of a mesophilic protein can be improved relatively easily [12,13]. For example, Németh and colleagues improved the T_m_ of a cellulose C by 3°C [12], and in our opinion this approach is significantly more effective than well-established experimental approaches such as library screening and random site-directed mutagenesis.

Despite the effectiveness of this method, applications of it are often hampered. First, it requires alignment of the target mesophilic protein sequence with those of thermophilic homologs, but not every mesophilic protein has a thermophilic homolog. Even though one can find the sequences from corresponding thermophilic proteins, the design accuracy remains limited owing to a lack of structural information. Although structure prediction has become a routine step during protein engineering, it requires a very skillful computational biologist to perform this well. Second, not all favorable features of a thermophilic protein can be incorporated into the structure of a mesophilic protein, as the function of the target protein must remain intact. In industry, even so-called ‘thermophilic’ proteins are not stable enough for industrial applications, and in this situation it is difficult to create a more thermostable protein from a mesophilic one. Therefore, creating an industrially applicable protein on the basis of the thermophilic protein could prove advantageous. To attain this objective, experimental biologists usually utilize well-established methods including library screening and random site-directed mutagenesis. Although effective, these methods can be time-consuming and costly. Furthermore, traditional methods such as library screening can result in researchers obtaining the same sequence time and time again, owing to limited evolutionary selection pressure. With the development of molecular modeling theory and computer science, several rational design approaches have been developed, and rational designs have been progressively applied as a routine step during protein thermostability engineering. However, the success rate for purely rational design is largely discounted because of the lack of structural information and design experience. Therefore, the question of whether it is possible to learn something from those mesophilic proteins to improve the thermostability of thermophilic proteins further must be addressed.

Amylosucrase (AS) is a type of glucosyltransferases (E.C. 2.4.1.4) that belongs to the Glycoside Hydrolase (GH) Family 13. In the presence of an activator polymer, *in vitro*, AS can catalyze the synthesis of an amylose-like polysaccharide composed of only α-1,4-linkages, using sucrose as the lone energy source, making it a potential candidate for industrial applications. Other enzymes responsible for the synthesis of such amylose-like polymers require the addition of expensive nucleotide-activated sugars. Despite its potential, the industrial development of AS is limited owing to its weak thermostability. During this study, we wished to compare the structure of *Deinococcus geothermalis* amylosucrase (DgAS), the most thermostable amylosucrase identified to date, with those of mesophilic amylosucrases, namely *Neisseria polysaccharea* amylosucrase (NpAS), *Deinococcus radiodurans* (DrAS) and the newly determined *Arthrobacter chlorophenolicus* (AcAS) [14], in the hope of further improving its thermostability. Using these mesophilic homologs, we have identified some promising substitutions, and believe these designs are helpful for improving the thermostability of DgAS.

## Materials and methods

### Modeling

DgAS and NpAS are the only two ASs whose structures have been experimentally determined, and their crystal structures can be accessed via the protein data bank (PDB, http://www.rcsb.org/). During this work, PDB files of 3UCQ [15] and 1G5A [16] were chosen for DgAS and NpAS, respectively, as in our previous work [17] and for the same reason. These two files were processed before further analysis, following the procedures outlined in [17]. In relation to DrAS and the newly identified AcAS, the powerful modeling program I-TASSER [18,19] was used. All parameters of I-TASSER were kept at default settings. The sequence identities between DrAS and NpAS/DgAS were 41% and 74%, respectively. For AcAS, the values were 57% and 44%.

### Structure and sequence comparison

With the aim of discovering how to improve the thermostability of DgAS, the structure of DgAS was superimposed on to those of NpAS, DrAS and AcAS using the “Align and Superimpose Proteins” protocol provided by Discovery Studio (DS). Details concerning the comparisons between the structure of DgAS and those of the mesophilic homologs are described in the Results section.

Sequence alignment was not arbitrarily performed using the BLAST program [20]; we utilized structural similarity and human expertise during the sequence aligning process to correct the alignment generated by automatic methods.

### H-bonds and salt-bridges

H-bonds and salt-bridges are important contributors to protein thermostability. Properly incorporating an H-bond or a salt-bridge (usually on the protein surface) can improve thermostability. These two values are calculated by VMD [21]. The distance (between the donor and the acceptor atoms) and angle (donor-hydrogen-acceptor) cutoff for a H-bond were set to 3.0 Å and 150°, respectively. The distance cutoff for a salt bridge was set to 3.5 Å.

### Contact order

The contact order of a protein is a measure of the locality of the inter-amino acid contacts in the protein’s native state tertiary structure. It has been demonstrated to be a major determinant of protein thermostability and is calculated as the average sequence distance between residues that form native contacts in the folded protein divided by the total length of the protein. Higher contact orders indicate longer folding times [22,23], and low contact order has been suggested as a predictor of potential downhill folding, or protein folding that occurs without a free energy barrier [24]. This effect is thought to be due to the minor loss of conformational entropy associated with the formation of local, as opposed to nonlocal, contacts [23]. Contact order (CO) is formally defined as:

$$\mathrm{CO}=\frac{1}{L\cdot N}\sum^{N}\Delta L_{\mathrm{ij}}$$

Where *N* is the number of contacts between heavy atoms in the protein, *L* is the length of the protein in amino acid residues, and Δ*L*_*ij*_ is the number of residues separating the interacting pair of heavy atoms. Absolute contact order (Abs_CO) is defined as:

$$\mathrm{Abs}\_\mathrm{CO}=\frac{1}{N}\sum^{N}\Delta L_{\mathrm{ij}}=\mathrm{CO}\times L$$

Contact orders of four AS were calculated with default parameters by the contact order calculation server (http://depts.washington.edu/bakerpg/contact_order/) provided in Washington University.

### Contact density

In a previous study, we demonstrated that the value of “number-of-contacts-per-residue (NCPR)” is related to protein thermostability and unfolding order [17]. Herein, we rename NCPR ‘contact density’ for convenience. The contact density value corresponds directly to the compactness of a protein, which has been demonstrated to be critical for protein thermostability [25]. The method for calculating contact density has been described previously [17].

### Free energy calculation

Thermostability is strongly correlated to folding free energy. To evaluate the thermodynamic stability of wild type (WT) and mutant DgAS, the folding free energy changes were estimated utilizing the FoldX program [26-28], which uses a full atomic description of the structure of a protein. The predictive power of the FoldX force field has been tested on a very large set (more than a thousand) of point mutants spanning most of the structural environments found in proteins. Detailed descriptions of the energy function used by FoldX are addressed elsewhere [26-28].

### Local structure entropy (LSE)

Since structure conservation reflects the effects of intrinsically stable (context-independent) sequence patterns and long-range generic contributions (context dependent) from surrounding residues [29], structural entropy provides a convenient structural measure of thermostability. The LSE value of a protein is closely related to its intrinsic thermostability; in general, thermostable proteins have smaller LSE values than their mesophilic homologs. For detailed descriptions of the LSE method, please see Chan’s original work [30]. In the current LSE was calculated for DgAS and its mutants using a JAVA program (in house script).

## Results

### Modeling and validation of the structures of DrAS and AcAS

The structures of AcAS and DrAS were modeled using I-TASSER, based on the structural information relating to DgAS and NpAS. Models were sorted according to their C-scores, and the one with the best C-score were selected as the final model (Additional file 1: Figure S1, structural models). The C-scores of the final models for DrAS and AcAS are 1.39 and 1.02, respectively. The C-score is a confidence score for estimating the quality of predicted models by I-TASSER, and typically ranges from −5 to 2. A higher C-score signifies a model that can be regarded with confidence. Subsequently, the final structural models of AcAS and DrAS were evaluated using Ramachandran plots [31,32] and Profiles-3D [33].

According to the final models produced by I-TASSER, only 1.1% and 1.0% of the residues in AcAS and DrAS, respectively, are located in the disallowed regions of the Ramachandran plot. In comparison, 0.2% and 0.1% residues of DgAS and NpAS, respectively, are located in the disallowed regions. On the basis of this comparison, the models of AcAS and DrAS are comparable in overall quality with the experimentally determined structures of DgAS and NpAS, at least in terms of backbone conformations.

The Profiles-3D method measures the compatibility of an amino acid sequence with a 3D protein structure. The method can be used to check the validity of a hypothetical protein structure by measuring its compatibility with the sequence of that protein. The VERIFY score of the protein given by a Profile-3D run is a useful measure of the overall quality of the structure. Proteins of similar size are expected to give similar high and low VERIFY scores. If the model structure has a VERIFY score higher than or close to the expected high score, the overall model is likely to be correct. The VERIFY scores of the four ASs are listed in Table 1. As they are of very similar size, the expected high and low scores for AcAS and DrAS were comparable. According to Table 1, the overall quality of the structural models of AcAS and DrAS can be used confidently as references for subsequent design trials. The VERIFY score for the model of DrAS is higher than that of AcAS. This is expected, as the sequence identities between DrAS and templates are higher than those between AcAS and templates.

**Table 1:** VERIFY scores for the four AS structures

	**High score**	**Low score**	**VERIFY score**
DgAS	297.61	133.93	327.35
DrAS	294.84	132.68	290.05
NpAS	287.46	129.36	318.87
AcAS	294.38	132.47	268.52

### Structural comparison between DgAS and mesophilic homologs

The structures of DgAS and its mesophilic homologs were compared to gain insight into their differences and to discover rules important for thermostability design. The structure of DgAS was superimposed on to those of its mesophilic homologs and the root-mean-squared deviation (RMSD) between DgAS and the other ASs were calculated as 0.64, 2.38 and 1.47 Å (in the sequence DrAS, NpAS and AcAS). On the basis of this structure superimposition, sequence alignment (Additional file 2: Figure S2) for these ASs was adjusted accordingly to improve the design accuracy.

Comparing a protein with several homologs in great detail is extremely laborious, particularly when several deletions/insertions exist. We, therefore, will just address major differences here.

**Figure 1:**
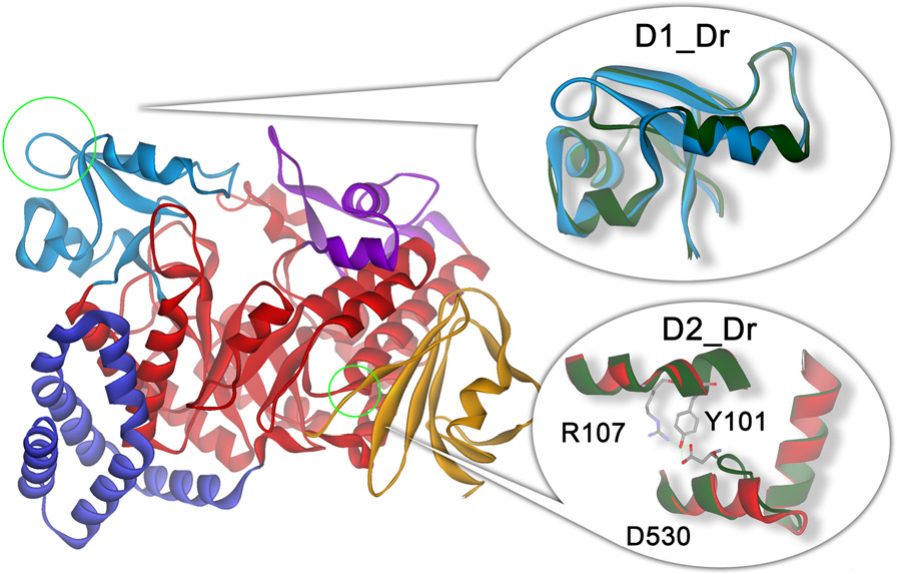
**The major structural differences between DgAS and DrAS.** The N, A, B, B’ and C-domains of DgAS are colored deep blue, red, sky blue, purple and orange, respectively. In the two inset figures, the local structures of DrAS are superimposed on to the corresponding parts of DgAS and the local structure of DrAS are colored deep green for clarity.

Visual inspection of each aligned region and the corresponding RMSD demonstrated that the structure of DrAS is similar to that of DgAS. Given that the structural model is correct, DgAS and DrAS differ in only two small regions (see the insets of Figure 1). D1_Dr (abbreviation for difference region 1 in DrAS) is located in the B-domain, and is attributable to the four-residue deletion in the corresponding position of the DrAS sequence (corresponding to _237_GEGE_240_ in DgAS). In relation to DrAS, the deletion relative to DgAS shortens the connecting loop between β1 (228–236 in DgAS) and the β-strand (243–248 in DgAS) of the B-domain and changes the loop into a sharp turn. In contrast, D2_Dr, which is located in the A-domain, results from the _530_DAATG_534_ insertion in DrAS, causinga bulge in the local structure of DrAS. Owing to this bulge, the side-chain of D530 interacts in several ways with R107 and Y110 (in α1 of A-domain, described in Ref [15]) through the hydrogen network formed among them. This hydrogen network is important for enhancing the connection between α1 and the loop of _530_DAATG_534_.

**Figure 2:**
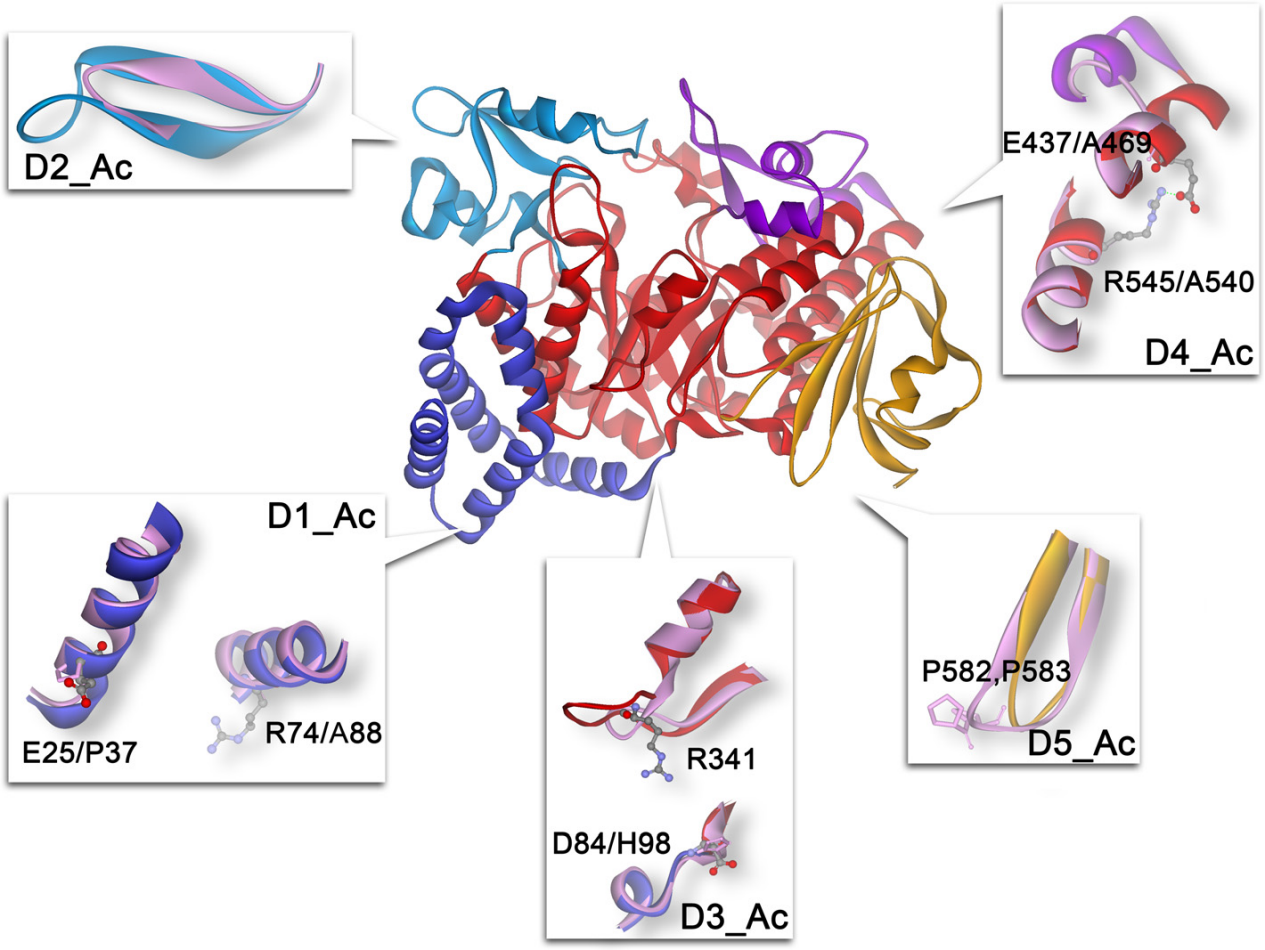
**The major structural differences between DgAS and AcAS.** DgAS is indicated by the same coloring scheme. The local structures of AcAS (pink) are superimposed on to the corresponding parts of DgAS.

Apart from the badly aligned N-terminal of the N-domain, AcAs differs from DgAS in five regions, and most of these differences are caused by insertions or deletions in the corresponding positions of AcAS (Figure 2). In terms of DgAS, E25 stands on an α-helix of the N-domain (no counterpart in AcAS because of deletion, D1_Ac), which can form a salt-bridge with R74 of another DgAS monomer [15]. However, this salt-bridge is not conserved in AcAS owing to deletion and substitution in corresponding positions. D3_Ac is located at the very bottom of the catalytic A-domain. The corresponding region of DgAS is an extended loop (_341_RAHHG_345_), which is involved in forming the homodimer interface. In particular, the salt-bridge formed between R341 of one monomer and D84 of another, together with that formed between R74 and E25, are critical for stabilizing the DgAS homodimer [15]. Therefore, AcAS is unable to form a dimer owing to lack of the extended loop and some pivotal salt-bridges.

D2_Ac is also located in the B-domain, closely resembling the conformation of D1_Dr. D4_Ac is situated at the joint between the C-terminal of the B’-domain and the N-terminal of the α7-helix of the A-domain. According to structure and sequence comparisons, corresponding regions of DgAS and DrAS are eight residues longer than those of AcAS. According to the crystal structure of DgAS, E473 forms a salt-bridge with R545 (α8), improving connections between α7 and α8. However, this salt-bridge does not exist in AcAS owing to deletion and substitution in corresponding positions. The last major difference (D5_Ac) between DgAS and AcAS lies in a beta-turn of the C-domain, where AcAS is three residues longer than DgAS. The proline-enriched elongated beta-turn could help to stabilize the local structure of AcAS by increasing rigidity.

Differences between the structures of DgAS and NpAS have been independently discussed elsewhere by Guérin [15] and Liu [17], and will not be elaborated on within this paper.

### Factors critical for thermostability

Some generally accepted key factors for thermostability were calculated for the four ASs to analyze structural differences that cannot be detected visually. Under no circumstances could we produce successful designs without knowing why DgAS is more stable than other ASs. It should be noted that homology models were used to calculate structural properties. Although the models of AcAS and DrAS were predicted carefully, we cannot guarantee that all calculations based on these models are correct.

#### Proline numbers

Proline residues have the most rigid structures among the 20 naturally-occurring amino acid residues, mainly because of the cyclic side-chain. Being a building block of a peptide or protein, the torsion angles (φ, ψ) of proline residues are usually restrained around two very narrow regions. In general, a proline residue reaches the configure energy minimum at approximately φ = −60°. Owing to its special configuration, a proline residue often acts as the connecting component between two regular secondary structures. The rigidity of proline residues allows them to hold the local structure together tightly and thereby decrease the conformational entropy of the unfolded state. Consequently, substituting a residue in a proper position with a proline residue can theoretically enhance the thermostability of a protein. The proline number and the corresponding distribution for each AS is calculated and presented in Table 2.

**Table 2:** The distribution of proline residues in the four ASs among each domain

	**Total**	**N**	**A**	**B**	**B’**	**C**
DgAS	36	2	20	6	2	6
DrAS	38	4	17	5	3	9
NpAS	30	4	16	5	2	3
AcAS	43	7	21	5	3	7

According to Table 2, proline numbers for these four ASs range from 30 to 43. Among these proline residues, only 17 are absolutely conserved. Surprisingly, AcAS has the most proline residues, whereas DgAS, the most stable AS identified so far, has only one more proline than NpAS. This demonstrates that the total proline number alone is not a good indicator of stability, a point stressed in our previous work [17], where we found that the proline number for each individual domain of the stable DgAS is not necessarily more than that of NpAS, although the total proline number of NpAS is six lower than that of DgAS. This provides the possibility of enhancing protein stability by substituting proper residues with proline residues based on less stable proteins. This point will be discussed further in the following section.

#### H-bonds and salt-bridges

Previously, we have provided details relating to the H-bonds and salt-bridges of DgAS and NpAS, and in the current study we calculated these properties for the four ASs using the new models. The details for all ASs analyzed in this work are presented in Table 3.

**Table 3:** H-bonds and salt-bridges of the four ASs

	**H-bonds (3.0Å, 150°)**			**Salt-bridges (3.5 Å)**
	**All**	**Back-Back**	**Side-Side/Back**	
DgAS	255	180	75	42
DrAS	239	138	101	39
NpAS	245	150	95	39
AcAS	243	120	123	35

DgAS has more H-bonds than any other AS, particularly in relation to backbone-backbone type H-bonds. It also has the most salt-bridges. Surprisingly, we discovered that DrAS and AcAS have fewer backbone-backbone H-bonds and salt-bridges than DgAS and NpAS. To minimize errors introduced by the structure modeling process, a series of rigorous refinements and minimizations were carried out, but the results were comparable. In addition to the modeling error, we propose that the large difference in H-bonds and particularly in salt-bridges could have resulted for other reasons.

During the current study we identified that DgAS has more charged residues than any other AS. Although DrAS has the second most charged residues, almost 20% of these are not conserved between DgAS and DrAS. From visual inspection it was determined that DrAS has more unpaired charged residues than DgAS, owing to their different locations; other than NpAS, AcAS has the fewest positively-charged residues. Although AcAS has the second highest number of negatively-charged residues, several are unpaired with positively-charged residues owing to their locations.

The electrostatic interactions of these four ASs were calculated with the CHARMm [34,35] module embedded in DS. On the basis these calculations, electrostatic interactions in DgAS are significantly stronger than in the other ASs (DgAS > DrAS, NpAS > AcAS). This implies that the strong electrostatic interactions in DgAS are partially responsible for its superior stability.

#### Contact order and contact density

The relative and absolute contact orders of these four ASs were calculated using a web server and the corresponding results are presented in Table 4. The four ASs have very similar *CO* and *Abs*_*CO*. Considering these AS share quite similar fold, this result just indicates they have similar folding rate. Previously, we have analyzed the contact density of DgAS and NpAS; herein, this property was revisited for each of the four ASs. DgAS was demonstrated to have the highest contact density. This could account, at least in part, for its superior stability. The contact densities of DrAS and AcAS were substantially lower than those of DrAS and NpAS. This result correlates with the fact that DrAS and AcAS have fewer backbone-backbone H-bonds (see Table 3). The relatively less-compact packing of NpAS, DrAS and AcAS could account for their relatively weak VDW interactions (Table 5).

**Table 4:** Contact orders and contact densities of the four ASs

	**CO**	**Abs_CO**	**Contact density**
DgAS	30.20	0.046	3.06
DrAS	30.88	0.048	2.88
NpAS	30.34	0.048	3.02
AcAS	30.00	0.047	2.86

**Table 5:** Potential energy and corresponding VDW and electrostatic contribution of the four ASs

	Potential energy*	**VDW**	**Electrostatics**
DgAS	−26894.41	−3128.98	−22366.96
DrAS	−23560.66	−2691.14	−17794.85
NpAS	−20901.96	−2874.80	−18032.24
AcAS	−21611.68	−2482.15	−16822.29

* all types of energy shown in this table are in units of kcal · mol^-1^.

### Design trials and computational validation

With the rapid development of theory concerning protein structure and computer science, the domain of protein design, or more specifically redesign, has grown increasingly prosperous. Several design tools and skills have been proposed over the past few decades. In this section, we have divided the design processes into three parts (protocols) and given representative cases for each design protocol. All designs were evaluated by free energy calculation and LSE.

#### The easy way: substitution of specific residues with proline residues

According to sequence alignments between DgAS and the other three ASs, 19 positions in DgAS are possible for proline substitution. However, scrutinizing the structural model of DgAS demonstrates that only 14 of them are located in the allowable region for proline residues. Further, changes in folding free energy and LSE values for each of these seemingly allowable substitutions were calculated using methods outlined above. The results are presented in Table 6.

**Table 6:** Mutants designed by the second protocol (X-Pro substitutions)

**Mutants**	**φ / ψ (°)**	**Secondary structure**^**a**^	**Domain**	**ΔΔG**_**f**_^**b**^	**ΔLSE**
E36P	−68.08/−43.76	H	N	−1.10	−0.0000352
A44P	−68.62/−30.86	H	N	3.21	−0.0002585
A70P	−51.82/−37.08	H	N	−2.01	−0.0002950
D113P	−62.44/−35.24	H	A	−1.00	0.0007323
R132P	−63.46/155.45	C	A	3.97	−0.0023640
N354P	−67.63/−36.53	H	A	0.41	0.0009455
A415P	−61.10/−41.68	H	B’	−1.55	−0.0000822
V438P	−67.20/134.06	E	B’	−0.50	0.0005602
I486P	−72.15/−31.78	H	A	5.03	0.0027557
V571P	−66.70/128.68	E	C	−0.79	−0.0013040
D585P	−74.44/147.85	C	C	−0.93	−0.0016631
T601P	−73.84/123.41	C	C	−0.25	−0.0013239
V608P	−56.44/−34.38	H	C	0.77	0.0017565
G637P	−46.17/129.60	T	C	−1.25	−0.0003485

^a.^ Letter H, E, C and T represent helix, sheet, coil and turn, respectively.

^b.^ kcal⋅mol^-1^.

**Figure 3:**
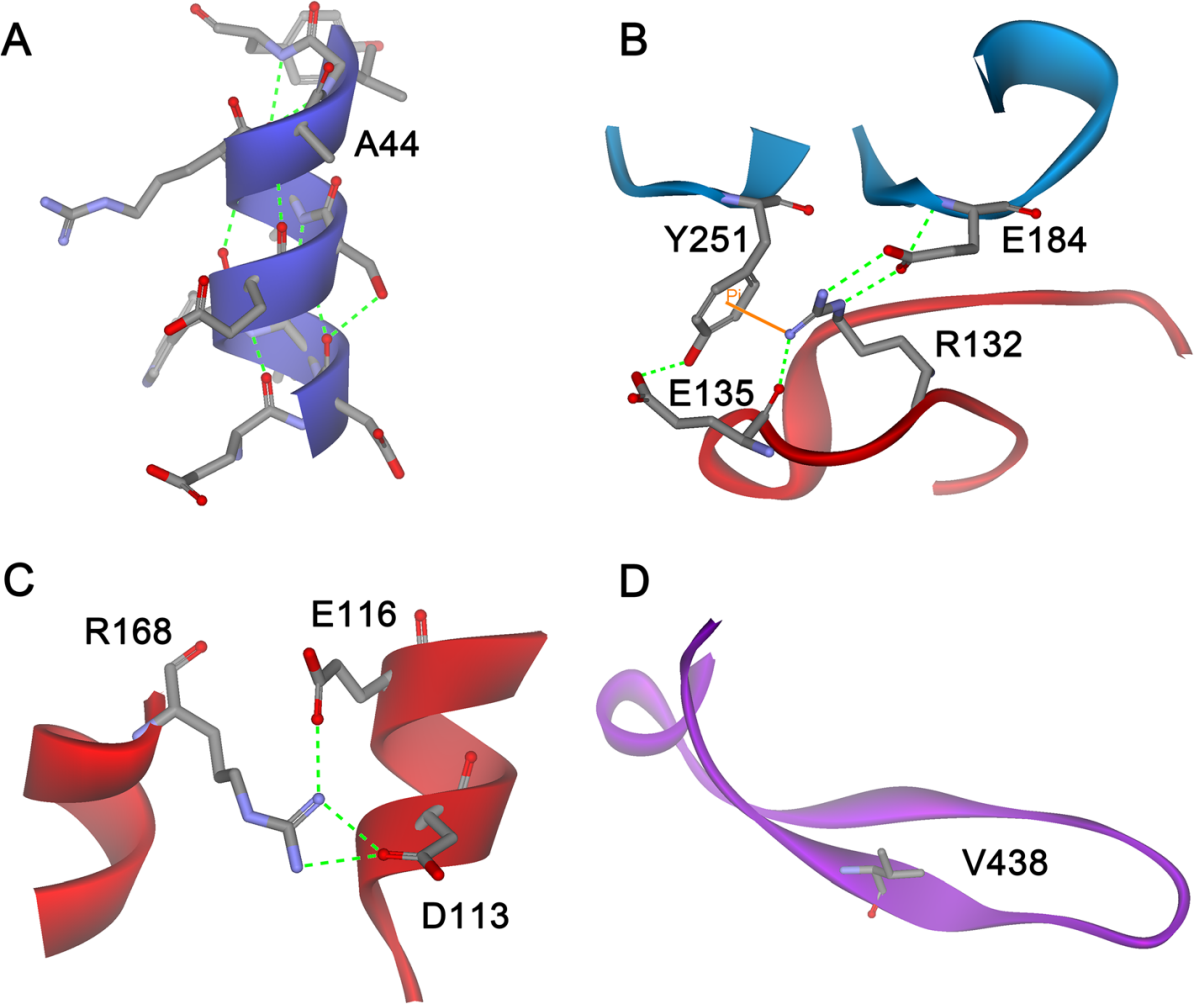
**The four exceptions in the X-to-proline type design (see the context for details).** The specific positions for A44, R132, D113 and V43 in DgAS are illustrated in **A**-**D**, respectively.

Among these substitutions, nine are independently predicted to be stabilized according to ΔΔG_f_ or ΔLSE. The results of ΔΔG_f_ fit well with those of ΔLSE; i.e., a negative ΔΔG_f_ corresponds to a negative ΔLSE. Only four exceptions were observed. A44 is located in the short α-helix_36-46_ of the N-domain, and A44P was predicted to be stabilized on the basis of its negative ΔLSE, whereas ΔΔG_f_ was not negative owing to VDW clashes caused by the A44P substitution. In view of its specific position, A44P could break well-established H-bonds (Figure 3A). Although R132P was predicted to be more stable than WT DgAS on the basis of its negative ΔLSE, the large increase in folding free energy could adversely affect this. According to the structure of DrAS, R132 is involved in forming H-bonds, salt-bridges and π-cation interactions with surrounding residues (Figure 3B). Although the R132P substitution can significantly decrease the conformational entropy of the unfolded state, it breaks the well-established interactions surrounding R132. Therefore, the total effect of this substitution in relation to folding free energy could adversely affect the stability of the protein. The other two exceptions, D113P and V438P, possess some adverse effects identified through visual inspection, but their ΔΔG_f_ are predicted to be negative. D113 is located in α1 of A-domain and its side-chain is fully accessible to solvent and forms salt-bridges with R168 in α2. Therefore, D113P not only affects the structure of α1 but also breaks these important salt-bridges (Figure 3C). V438 is situated at the center of a β-strand in the B’-domain, and a proline residue in this position will distort the two-strand β-sheet of the B’-domain (Figure 3D).

Empirically, substitutions with negative ΔΔG_f_ and negative ΔLSE are more likely to enhance the stability of the engineered protein than substitutions with either solely negative ΔΔG or negative ΔLSE (in-house data). Substitutions with ΔΔG_f_ > −0.5 kcal⋅mol^-1^ are excluded from further consideration for the reason explained in the part of the methods section concerning free energy calculation. According to these criteria, only six substitutions remain for subsequent experimental validation (not included in this paper). For the sake of clarity, all substitutions that passed all filters in each design trial were nominated as ‘promising substitutions’.

**Figure 4:**
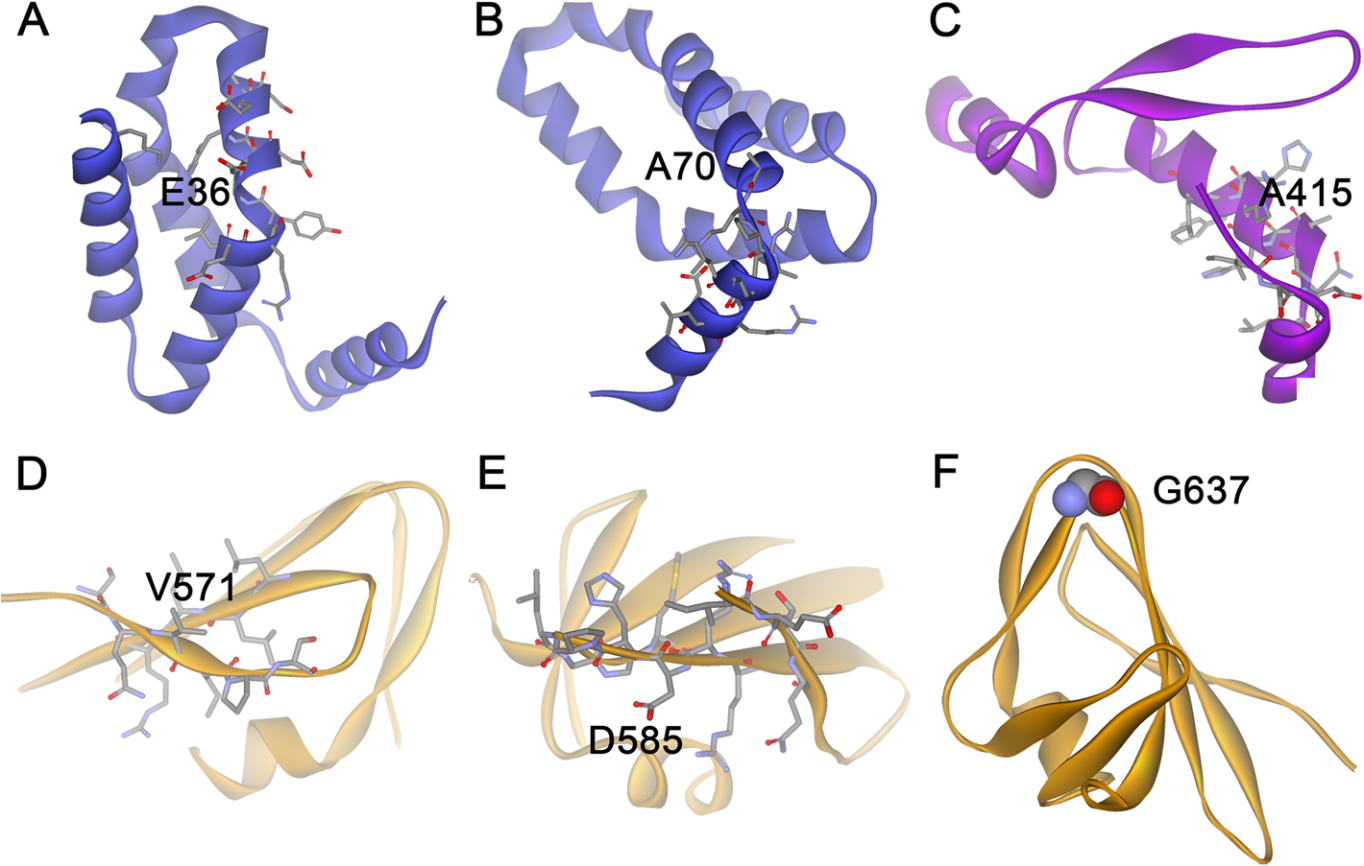
**The six promising X-to-proline substitutions.** The specific positions for E36, A70, A415, V571, D585 and G637 in DgAS are illustrated in **A**-**E**, respectively. In **F**, G637 is highlighted by the CPK model because of its small size.

Among the six promising substitutions, two are located in the N-domain, one in the B’-domain and three in the C-domain, and none are located in the conserved A-domain. This observation reflects the fact that the sequence and structure of the A-domain is crucial for the functions and stability of AS. According to Table 2, the N-domain of DgAS has the fewest proline residues and in the N-domain of DgAS, E36 and A70 are located at the N-terminals of α-helices (Figure 4A and B). Their side-chains all point outward and substituting them with rigid prolines would not result in obvious VDW clashes. In the other three ASs, positions corresponding to E36 of DgAS are taken up by proline residues. On the basis of our calculations, E36P and A70P contribute positively to stability. According to a previous analysis [17], the contact density of the DgAS N-domain is greater than the others. As far as residue size is concerned, proline is comparable with glutamate and bigger than alanine. Therefore, the contact density of the N-domain could be increased by the incorporation of the two proline residues, provided the overall fold does not change significantly. On the basis of these analyses, these two substitutions are probably helpful to the stability of DgAS. Ala415 is located at the N-terminal of the longest α-helix of the B’-domain, and no obvious steric hindrance is evident for the A415P substitution (Figure 4C). V571 and D585 are situated in β-strands of the C-domain (Figure 4D and E). Although no evidently adverse effects are detected, substituting V571 and D585 with proline residues could affect the well-established hydrophobic packing and the formation of local secondary structure. G637 lies at the sharp turn of two β-strands in the C-domain (Figure 4F). G637P substitution is encouraged, as the corresponding positions in the other three ASs are taken up by proline residues. Therefore, each of these promising substitutions should be included in subsequent experimental validations.

#### The confused way: should we substitute glycine residues with bigger ones or vice versa?

Glycine is the most flexible of the 20 naturally occurring amino acid residues. It is usually located at turns or loops in proteins, and can access larger conformational spaces than other residues. Because glycine is small, it is prone to facilitate the motions of local structures around it and increase the conformational entropy of any state. The unfolded state is an ensemble of several non-native states, so the overall effect of introducing a glycine residue into a protein should be unfavorable for stability. Since glycine is the only residue whose backbone can adopt φ > 0 with little steric hindrance, it should, as far as possible, occur in the right half of the Ramachandran plot. A glycine residue with negative φ should be replaced by other residues to elevate thermostability if the space around it is large enough. For the sake of convenience, we nominate the glycine residues with positive φ as φ^+^ glycine residues, and the ones with negative φ as φ^-^ glycine residues.

During the course of evolution, selection pressure has rendered the sequences and structures of native proteins optimal or thereabouts in terms of function and stability. However, if native proteins are nearly optimal, why are there still many seemingly unnecessary glycine residues in them? A likely explanation is that these “unnecessary” glycine residues are actually indispensable for the trade-off between function and stability. Previously, we identified only one dispensable glycine residue among the 49 in DgAS [17], indicating that several glycine residues are indispensable for the functioning and stability of this protein. However, substituting a normal (i.e. any except Gly and Pro) residue with glycine can improve the stability of the target protein. Previously, during stability engineering on a human-source antibody, we substituted an alanine residue located at a β-turn (whose φ is positive) with a glycine residue. The half-inactivation temperature of the engineered antibody was elevated by 1.2°C (unpublished data). In the current study we attempted to substitute residues with positive φ with glycine residues.

We used the structures of the other three ASs for reference, and only one possible position (A502) was determined. In contrast to proline residues, glycine residues are not overly conserved. In other words, they appear to occur at random positions. Therefore, it is difficult to design similar φ^+^ glycine residues on the basis of homologous sequences. We identified all φ^+^ non-Gly residues in DgAS and determined whether it was feasible to substitute them with glycine residues. Detailed design examples are listed in Table 7.

**Table 7:** Mutants designed by the second protocol (X-Gly substitutions)

**Mutants**	**φ / ψ (°)**	**Secondary structure**	**ΔΔG**_**f**_	**ΔLSE**
D137G	52.24/47.93	C	2.04	0.0000340
A285G	54.46/27.26	C	−1.58	0.0007774
C297G	61.70/32,42	C	−0.77	0.0009561
N299G	63.66/31.87	C	−1.85	0.0016393
R341G	45.59/43.05	C	−0.32	0.0000000
R367G	55.60/29.07	C	0.86	0.0009665
Q440G	56.47/45.38	C	1.91	0.0004131
A502G	53.29/43.57	C	−0.24	0.0024813
Y638G	64.12/13.38	T	3.97	0.0007899

As Table 7 demonstrates, all possible substitutions are located in coils or β-turns. Surprisingly, LSE results suggested all substitutions have an adverse effect on stability. This may be attributed, at least in part, to the statistical nature of the LSE method. The LSE method only takes the local sequence (4 residues) into consideration despite proteins being three-dimensional. Long-range contacts are always observed, not only in large, multi-domain proteins but also in many small proteins. To avoid this defect, we propose that the LSE method should be used together with structure-based methods when possible.

**Figure 5:**
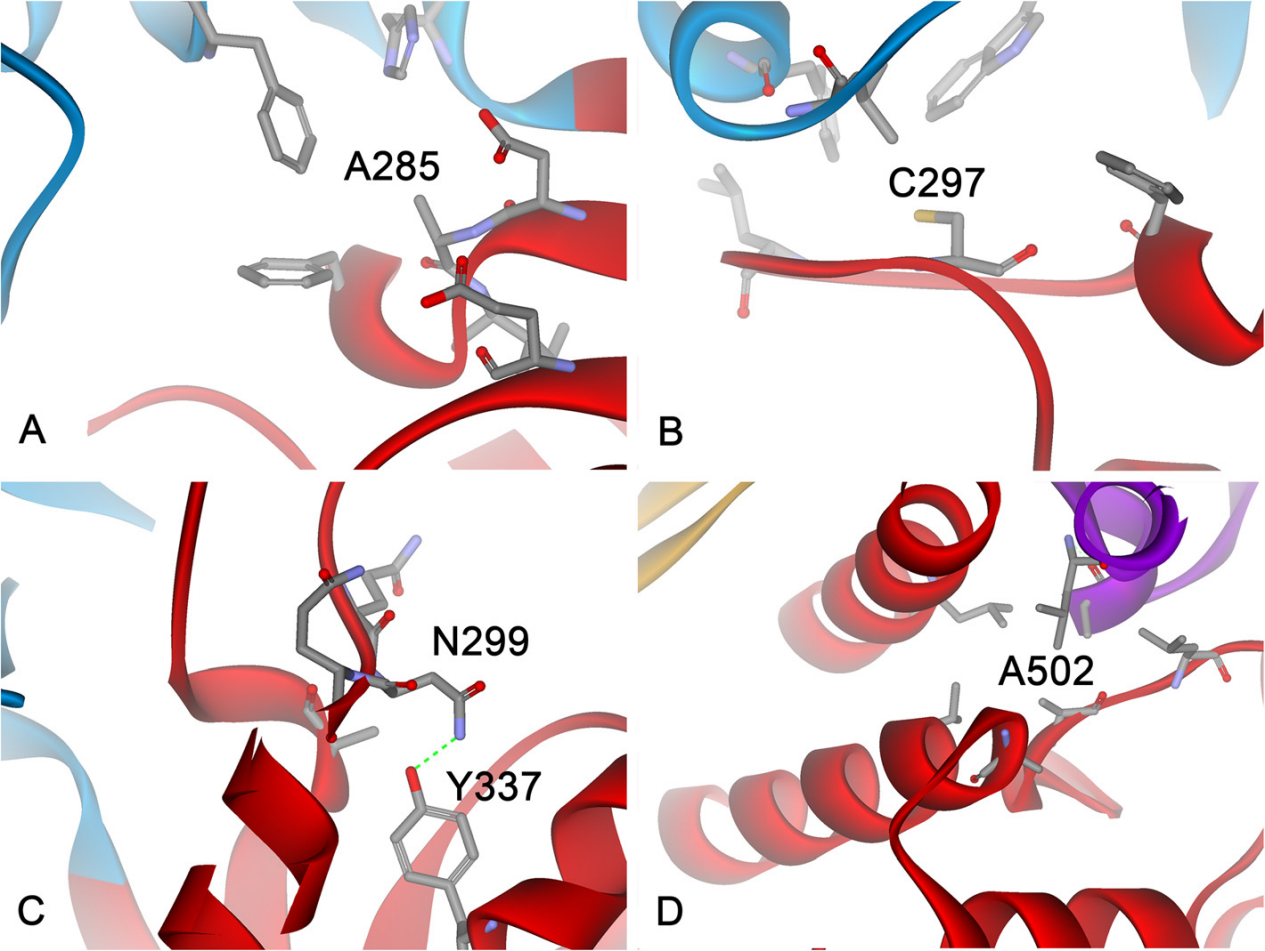
**The four promising X-to-glycine substitutions.** The specific positions for A285 (**A**), C297 (**B**), N299 (**C**) and A502 (**D**).

Five substitutions contribute to stability but only three, A285G, C297G and N299G, cause meaningful changes in folding free energy. Calculating the detailed energy decompositions of all substitutions demonstrated that VDW clashes for each of them are significantly decreased. For most small residue-to-Gly substitutions, adverse effects such as losing H-bonds and increased entropy in the unfolded state can be compensated for by decreased VDW clashes. For larger residue substitutions, decreased VDW clashes are normally not sufficient to compensate for the increased entropy and decreased VDW and/or electrostatic interactions. For example, R367 forms multiple H-bonds and salt-bridges with surrounding residues (Additional file 3: Figure S3). Therefore, substituting it with a glycine residue will break these favorable interactions. Although A285 is buried inside DgAS, it appears that substituting it with glycine does not significantly affect the surrounding VDW packing (Figure 5A). C297G is chosen in part because of a better solvation effect (Figure 5B). N299 is partially buried by surrounding polar residues, and only forms one H-bond with Y337 (Figure 5C). It is likely that N299G breaks the H-bond, but it also decreases the unfavorable desolvation energy caused by unsaturated H-bond donors in N299. A502G was the only residue designed on the basis of homologous sequences (Figure 5D). A502 and surrounding residues constitute a hydrophobic core. Although A502G decreases VDW clashes arising from the unfavorable backbone conformation, it also decreases the favorable VDW interactions and hydrophobic effect. Therefore, the effect of this substitution is to stabilize the target protein marginally.

#### The moderate way: enhance interactions among target residues

Interactions among residues in a protein predominantly concern VDW, electrostatic (including H-bond, salt-bridge and helix-dipole) and hydrophobic effects. In principle, stronger interactions can be achieved by substituting less-favorable residues with favorable ones. Almost all types of interaction are closely inter-correlated, as peptide chains in proteins are packed in a compact manner. In most cases, it is difficult to enhance one kind of interaction without affecting others. Although one can resort to automatic virtual scanning skills, which have become routine tools in several protein programs, the accuracy of this type of design is hampered by the inaccuracy of the original model and the rigid-backbone-based algorithm [36-38]. To overcome this defect, the local structure near the target position can be taken into consideration and the three less-stable enzyme structures can be used as references. On this basis, we propose a modified virtual scanning method. First, a virtual library was constructed on the basis of sequences of the four ASs; this initial library contained only residue types that occur at the target and the reference sequences. Secondly, residues sharing similar size and/or physicochemical characteristics with the WT residues were added to the library to improve diversity. Thirdly, the semi-saturation library was subjected to a standard scan protocol, although a negative design can be applied for better performance if the standard protocol fails. According to this automatic semi-saturation scan, numerous allowable substitutions were identified for DgAS. Checking structural models of these mutants identified that almost all mutants were stabilized by a combined mechanism. Table 8 lists nine mutants as examples.

**Table 8:** Mutants designed by the third protocol (enhancing interactions)

**Mutants**	**ΔΔG**_**f**_	**ΔLSE**	**Stabilizing forces**
A96V	−0.92	−0.0016646	VDW
A285R	−1.38	0.0016377	VDW, Electrostatics
A287K	−0.97	0.0011746	VDW, Electrostatics
S355L	−2.64	0.0000178	VDW, hydrophobic
A378S	−0.92	0.0015674	Electrostatics
N413D	−3.28	0.0003137	Dipole
A415E	−2.16	−0.0004484	VDW, Electrostatics
V444Q	−0.91	−0.0000552	Electrostatics
D499L	−3.00	−0.0019341	VDW, hydrophobic

**Figure 6:**
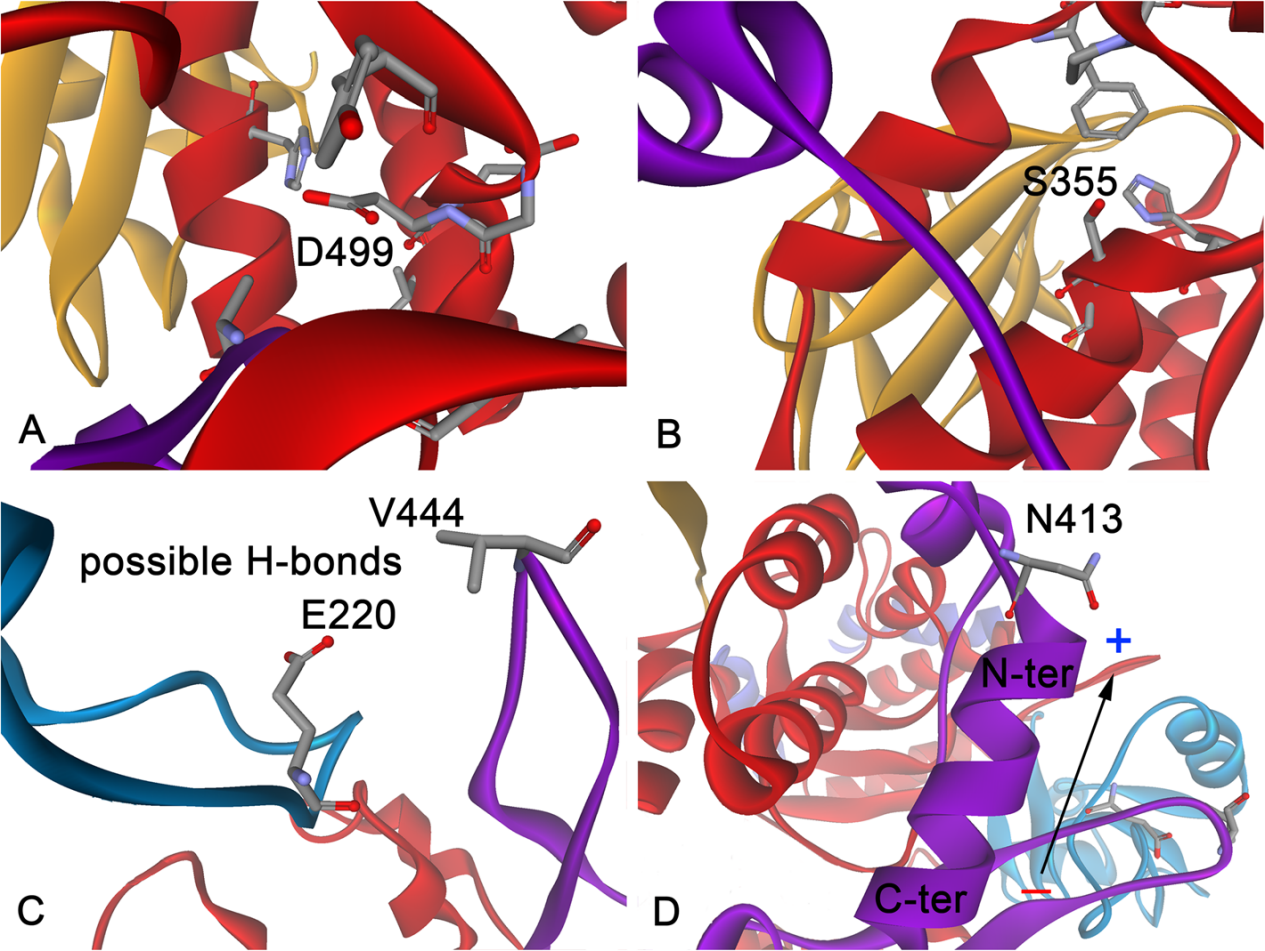
**Examples for the third type design.** The specific positions for D499, S355, V444 and N413 in DgAS are illustrated in **A**-**D**, respectively.

In principle, substituting small residues with bigger ones will enhance VDW interactions between the target residue and surrounding ones, but this does not imply that all small residues can be replaced with large ones, as larger residues also increase the likelihood of bad VDW clashes. Only careful substitutions will aid stability. As shown in Table 8, the stability of DgAS can be elevated by enhancing VDW interactions. Sometimes, polar residues are buried or partially buried with one or more unsaturated H-bond donors and/or acceptors. In this case, substituting them with proper hydrophobic residues will ease the unfavorable desolvation energy caused by the buried side-chains of polar residues. This can be perfectly illustrated by D499L and S355L. On the basis of the DgAS crystal structure, D499 is buried deep inside the A-domain; moreover, its side-chain forms no H-bonds with surrounding residues (Figure 6A). According to calculations, D499L can not only significantly enhance VDW interactions with surrounding residues but also decrease the bad desolvation energy. Similarly, S355 is also deeply buried inside the A-domain. The hydroxyl in the side-chain of S355 does not form H-bonds with surrounding residues (Figure 6B). According to sequence alignment and structure comparison, the corresponding positions in NpAS and AcAS are taken up by the hydrophobic Leu. On the basis of scanning results, S355L was selected owing to its improved hydrophobic effect.

Enhancing electrostatic interactions among residues is important during stability engineering, and this can be achieved by increasing the number of H-bonds and salt-bridges. A285R, A287K, A415E and V444Q are good examples of this; V444Q (Figure 6C) was selected on the basis of sequences of the less-stable AS (the corresponding position in DrAS is Q436).

A helix has an overall dipole moment caused by the cumulative effect of individual dipoles from the well-ordered amide groups pointing along the helix axis. This can lead to destabilization of the helix through entropic effects. To ease this adverse effect, the N or C caps of an α-helix can be modified to compensate for the dipole caused by the periodic nature of the α-helix. N413 is the N-cap of α-helix_413-426_ in the B’-domain (Figure 6D), and substituting it with an aspartate residue can effectively neutralize the positively-charged N-terminal of the helix, thereby removing the unfavorable dipole.

ΔLSE for these selected substitutions were calculated. In this type of design, it was determined that the LSE results did not correlate well with the free energy calculation. Chan’s work [30] suggests that this lack of correlation between the two methods can be attributed, at least in part, to the statistical nature of the LSE method.

## Discussion

During this study, the sequence and structure of DgAS was compared with those of DrAS, NpAS and the newly identified AcAS, and it was discovered that DgAS has favorable structural properties that can, in part, account for its superior stability. First, DgAS has the highest contact density, reflecting its strong VDW interactions. Second, the electrostatic energy of DgAS is much greater than that of the other ASs, which can be largely attributed to its excessive number of backbone-backbone H-bonds and salt-bridges.

Several groups have demonstrated that incorporating structural elements of thermostable proteins into their mesophilic homologs can improve stability. However, few protein designers have tried to find useful structural elements from less-stable proteins. This work not only focused on identifying allowable substitutions for DgAS stability engineering, but attempted to utilize useful structural elements from the other three ASs. By comparing the structure and sequence of DgAS with those of the others, promising substitutions were identified. In the first design trial (proline design), we attempted to introduce additional proline residues into the DgAS sequence, using the sequences of the other three ASs as references. Subsequently, in the second design trial (glycine design), the comparative design method was employed together with an empirical method to determine more designable positions. Finally, during the third design trial, we took advantage of automatic semi-saturation scanning and identified more allowable substitutions. On the basis of our analyses, some structural elements of less-stable proteins are better than their counterparts in the stable protein. This is not surprising, as a protein of 600 amino acid residues, theoretically, has an astronomical number (10^780^) of possible combinations. Therefore, even evolutionary selection pressure cannot guarantee that the most stable protein is constituted by the best structural elements. It is because of this that can we further improve the stability of the stable DgAS by utilizing structural elements from less-stable ASs. Given our experience of protein design (the author once designed hundreds of mutants for elevating stability, and the total accuracy was more than 40%), we believe several of these selected substitutions could enhance the stability of DgAS. All mutants shown here will be validated by subsequent experiments, the results of which will be presented in the near future.

In conclusion, we found that it is possible to stabilize a protein from thermophilic bacteria further by incorporating structural elements from less-stable proteins. On the basis of this work, it appears that the X-to-proline method can be easily integrated with information from other homologs. For proteins with few allowable positions for proline residues, the semi-saturation scanning method would be suitable. Although the glycine substitution method is not as effective as the other two, it could complement other methods. In addition to the design protocols mentioned above, semi-reasonable methods such as peptide fragment substitutions or domain swaps could also be used. However, the applications of these methods are limited, as protein engineers run the risk of impairing the functions and/or expression levels of the target protein. From our experience of daily design, a very complicated combination of single-site mutants is much more effective than the seemingly simple peptide fragment substitution method.

## Competing interests

The authors declare that they have no competing interests.

## Authors’ contributions

ML, HH, and JS carried out the experiments and drafted the manuscript. ML and JS programmed the GNM script. ML and HH designed this work. All authors read and approved the final manuscript.

## Supplementary Material

Additional file 1 Structural models of DgAS, DrAS, NpAS and AcAS. Click here for file

Additional file 2 The sequence alignment of DgAS, DrAS, NpAS and AcAS. Based on conservation, residues are colored by black, dark gray and gray, respectively. Click here for file

Additional file 3 R367 and surrounding residues of DgAS. According to its 3D-structure, R367 forms multiple H-bonds and salt-bridges with surrounding residues. Click here for file
