# Supplementary figures and images for: Is it possible to stabilize a thermophilic protein further using sequences and structures of mesophilic proteins: a theoretical case study concerning DgAS
Source: Theor Biol Med Model. 2013 Apr 10;10:26. doi: 10.1186/1742-4682-10-26 (PMC3639903; doi:10.1186/1742-4682-10-26)

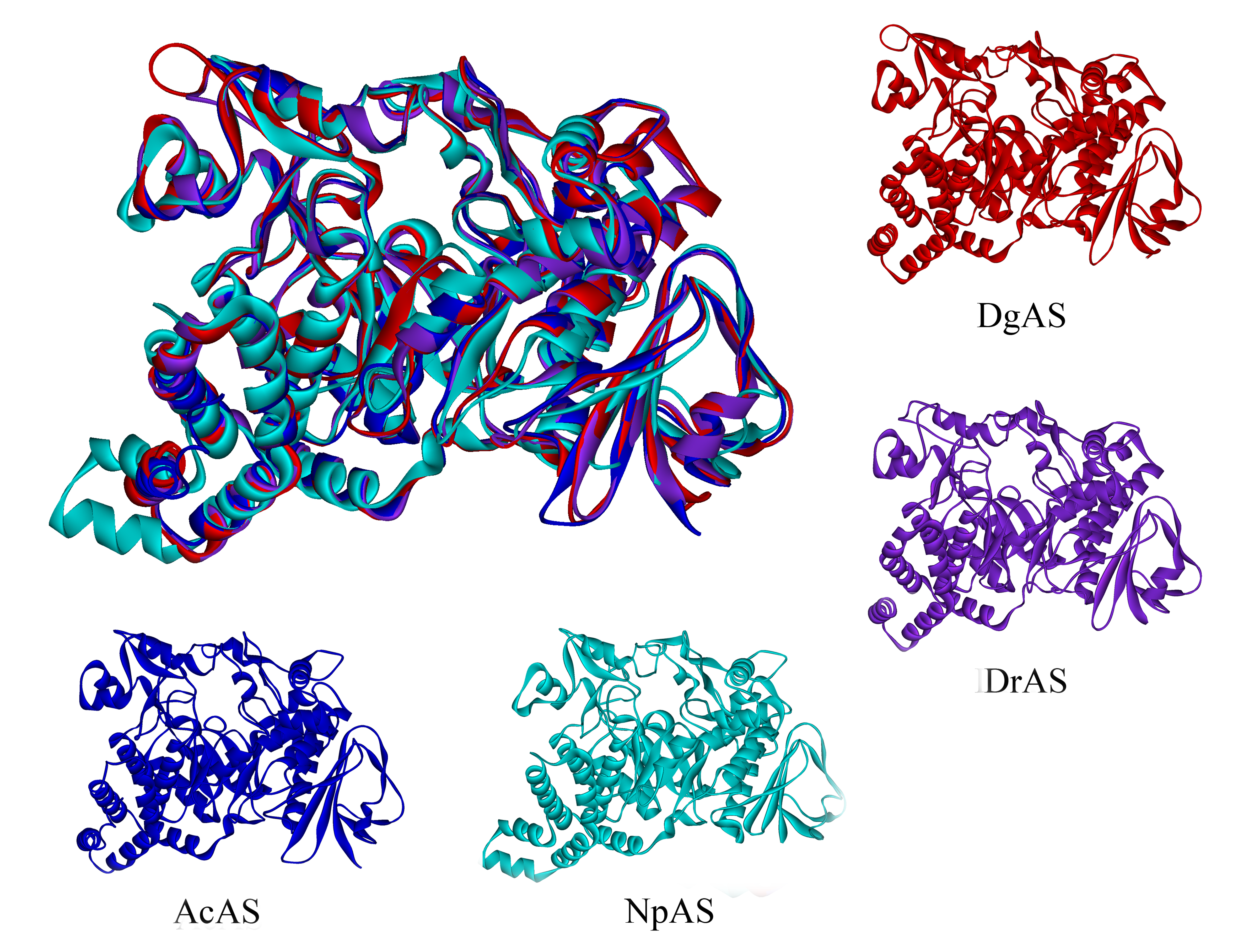

Supplement: Additional file 1 — Structural models of DgAS, DrAS, NpAS and AcAS. [file 1742-4682-10-26-S1.tiff]

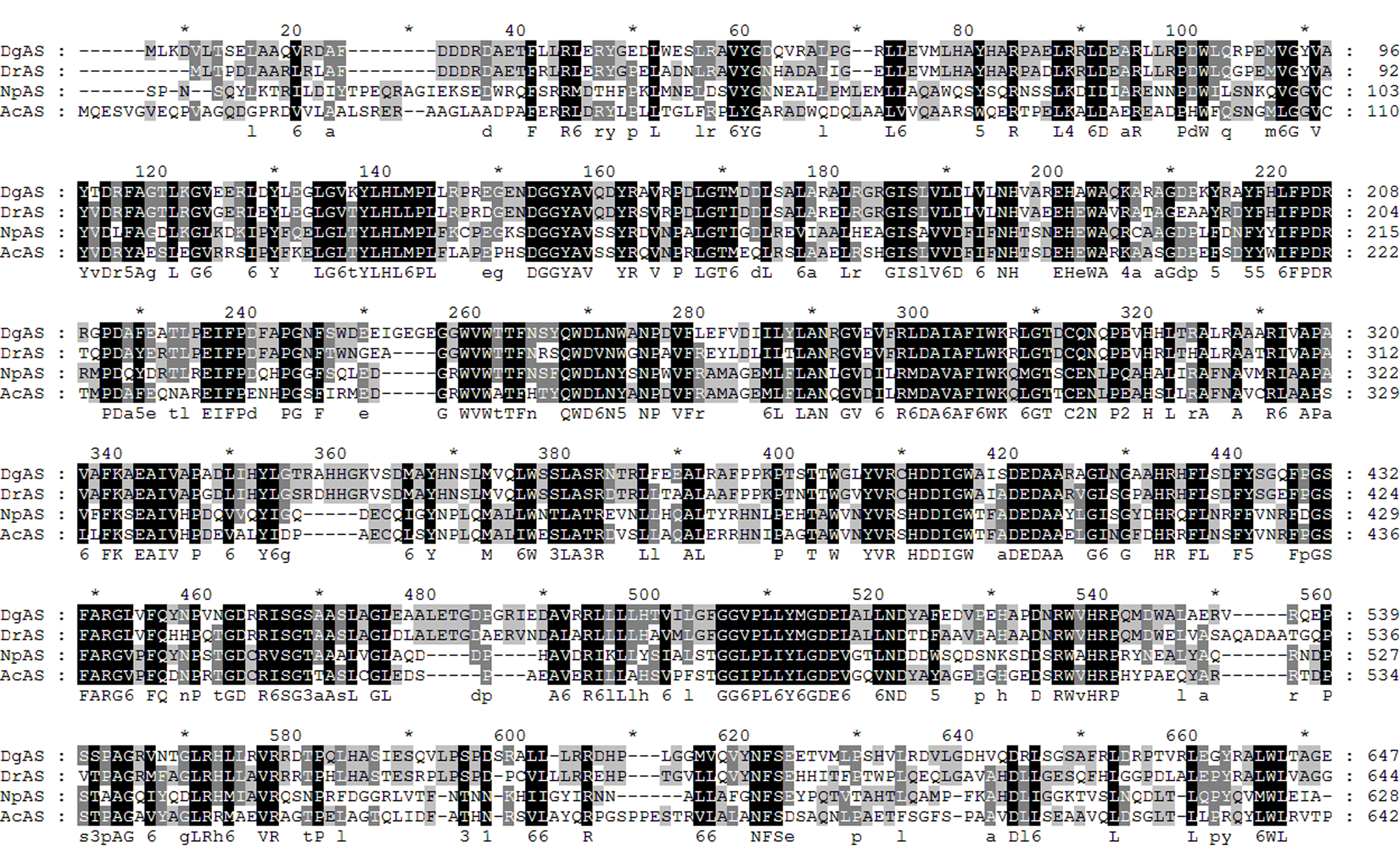

Supplement: Additional file 2 — The sequence alignment of DgAS, DrAS, NpAS and AcAS. Based on conservation, residues are colored by black, dark gray and gray, respectively. [file 1742-4682-10-26-S2.tiff]

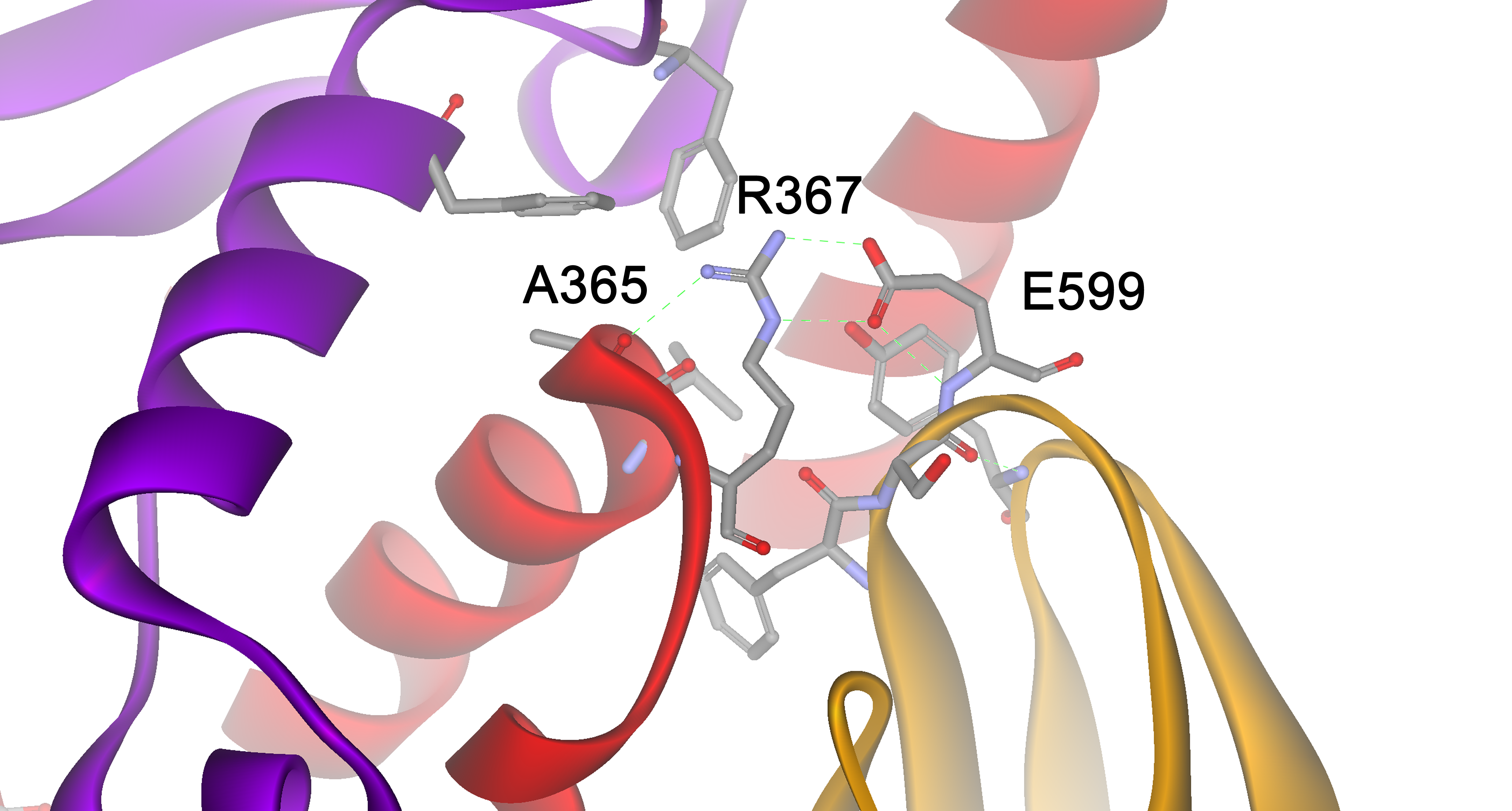

Supplement: Additional file 3 — R367 and surrounding residues of DgAS. According to its 3D-structure, R367 forms multiple H-bonds and salt-bridges with surrounding residues. [file 1742-4682-10-26-S3.tiff]
